# Presymptomatic cortical thinning in familial Alzheimer disease: A longitudinal MRI study

**DOI:** 10.1212/WNL.0000000000003322

**Published:** 2016-11-08

**Authors:** Philip S.J. Weston, Jennifer M. Nicholas, Manja Lehmann, Natalie S. Ryan, Yuying Liang, Kirsty Macpherson, Marc Modat, Martin N. Rossor, Jonathan M. Schott, Sebastien Ourselin, Nick C. Fox

**Affiliations:** From the Dementia Research Centre (P.S.J.W., J.M.N., M.L., N.S.R., Y.L., K.M., M.M., M.N.R., J.M.S., N.C.F.), UCL Institute of Neurology; Transitional Imaging Group (M.M., S.O.), Centre for Medical Image Computing, University College London; and London School of Hygiene and Tropical Medicine (J.M.N.), UK.

## Abstract

**Objective:**

To identify a cortical signature pattern of cortical thinning in familial Alzheimer disease (FAD) and assess its utility in detecting and tracking presymptomatic neurodegeneration.

**Methods:**

We recruited 43 FAD mutation carriers—36 *PSEN1*, 7 *APP* (20 symptomatic, 23 presymptomatic)—and 42 healthy controls to a longitudinal clinical and MRI study. T1-weighted MRI scans were acquired at baseline in all participants; 55 individuals (33 mutation carriers; 22 controls) had multiple (mean 2.9) follow-up scans approximately annually. Cortical thickness was measured using FreeSurfer. A cortical thinning signature was identified from symptomatic FAD participants. We then examined cortical thickness changes in this signature region in presymptomatic carriers and assessed associations with cognitive performance.

**Results:**

The cortical signature included 6 regions: entorhinal cortex, inferior parietal cortex, precuneus, superior parietal cortex, superior frontal cortex, and supramarginal gyrus. There were significant differences in mean cortical signature thickness between mutation carriers and controls 3 years before predicted symptom onset. The earliest significant difference in a single region, detectable 4 years preonset, was in the precuneus. Rate of change in cortical thickness became significantly different in the cortical signature at 5 years before predicted onset, and in the precuneus at 8 years preonset. Baseline mean signature thickness predicted rate of subsequent thinning and correlated with presymptomatic cognitive change.

**Conclusions:**

The FAD cortical signature appears to be similar to that described for sporadic AD. All component regions showed significant presymptomatic thinning. A composite signature may provide more robust results than a single region and have utility as an outcome measure in presymptomatic trials.

There is great interest in testing potential disease-modifying treatments for Alzheimer disease (AD) prior to the onset of symptoms.^1,2^ In order to facilitate this, robust and sensitive methods are needed to identify at-risk individuals, stage their disease, and track progression.^3,4^

Studies of volumetric MRI, using automated cortical thickness measurement, have suggested that sporadic AD is associated with cortical thinning in a relatively consistent pattern, or “signature,” of cortical regions.^5,6^ A composite signature is likely to be more robust to interindividual variation than using a single region alone. We were interested in seeing if a similar cortical signature pattern of loss could be used to track changes presymptomatically in autosomal dominant familial AD (FAD).

FAD shares many features, both pathologically and clinically, with the more common sporadic form of the disease.^7^ FAD genetic mutation carriers have relatively predictable ages at onset,^8,9^ which provides opportunity for prospective longitudinal study of individuals prior to the onset of clinical AD. Previous studies identified reductions in brain volumes before symptom onset, with longitudinal rates of change appearing more sensitive than cross-sectional measurement.^10,11^

We report the results of a prospective longitudinal study of cortical thickness in FAD mutation carriers. We first describe the identification of a cortical signature of FAD, determined in symptomatic participants, and then assess the utility of this approach in detecting presymptomatic change, in terms of both absolute thickness and rates of thinning. We then assess if baseline thickness can predict subsequent thinning and its correlation with preclinical cognitive changes.

## METHODS

### Standard protocol approvals, registrations, and patient consents.

The study was approved by the local research ethics committee and all participants provided written informed consent.

### Participants.

We recruited 85 participants between 2009 and 2014 to an ongoing longitudinal study of FAD at the Dementia Research Centre, University College London (appendix e-1 at Neurology.org). Forty-three participants were carriers of pathogenic mutations in either the presenilin 1 (*PSEN1*) (n = 36) or amyloid precursor protein (*APP*) (n = 7) genes and 42 were healthy controls (including 15 gene-negative siblings of the mutation carriers). The mutation carrier group included a total of 14 different *PSEN1* mutations and 4 different *APP* mutations. At baseline, 20 of the mutation carriers were symptomatic and 23 were presymptomatic.

There were 58 participants (43 mutation carriers and the 15 noncarrier siblings) from families affected by FAD, with 28 different families represented. For these participants, genetic testing was performed to determine the presence or absence of a mutation. Genetic data were provided only to designated individuals performing the statistical analysis, thus ensuring that both the participants and the clinicians assessing them remained blind to their genetic status.

At each timepoint, approximately annually, participants underwent MRI, neurologic examination, detailed neuropsychological assessment, and assessment with the Mini-Mental State Examination (MMSE) and Clinical Dementia Rating scale (CDR). The neuropsychological test battery comprised measures of general intellectual functioning (Wechsler Abbreviated Scale of Intelligence); verbal and visual recognition memory (Recognition Memory Test [RMT] for words and faces); short-term memory (forward digit span); executive function (backwards digit span); naming (Graded Naming Test); calculation (Graded Difficulty Arithmetic Test); and visuoperceptual skills (object decision test from the Visual Object and Space Perception battery [VOSP]). All individuals identified a close informant who was interviewed separately to gain a collateral history. Individuals were defined as symptomatic if consistent symptoms of cognitive decline were reported by the participant or their informant and the CDR was >0. Estimated years to symptom onset (EYO) was calculated for the presymptomatic mutation carriers by subtracting the participant's current age from the age at which the affected parent first developed symptoms.

### MRI acquisition.

All participants at all timepoints were scanned on the same 3T S TIM Trio scanner using a 32-channel phased-array head coil. A sagittal 3D magnetization-prepared rapid gradient echo T1-weighted volumetric MRI (echo time/repetition time/inversion time = 2.9/2,200/900 ms, dimensions 256 × 256 × 208, voxel size 1.1 × 1.1 × 1.1 mm) was acquired.

### Image analysis.

We measured cortical thickness across the cortical mantle at approximately 300,000 vertices using FreeSurfer v5.30's (surfer.nmr.mgh.harvard.edu) cross-sectional pipeline. The procedure used by FreeSurfer for the surface construction has been described and validated previously.^12,13^ Two modifications to the standard FreeSurfer processing stream were undertaken: a locally generated brain mask was used for skull stripping and ventricular segmentations were added to the white matter mask to improve cortical segmentation. Cortical thickness was smoothed with a 20-mm full-width at half-maximum kernel. All cortical segmentations were visually inspected. Mean cortical thickness values were calculated for 34 parcellated cortical regions bilaterally.

### Identifying the FAD cortical signature.

**Figure 1:**
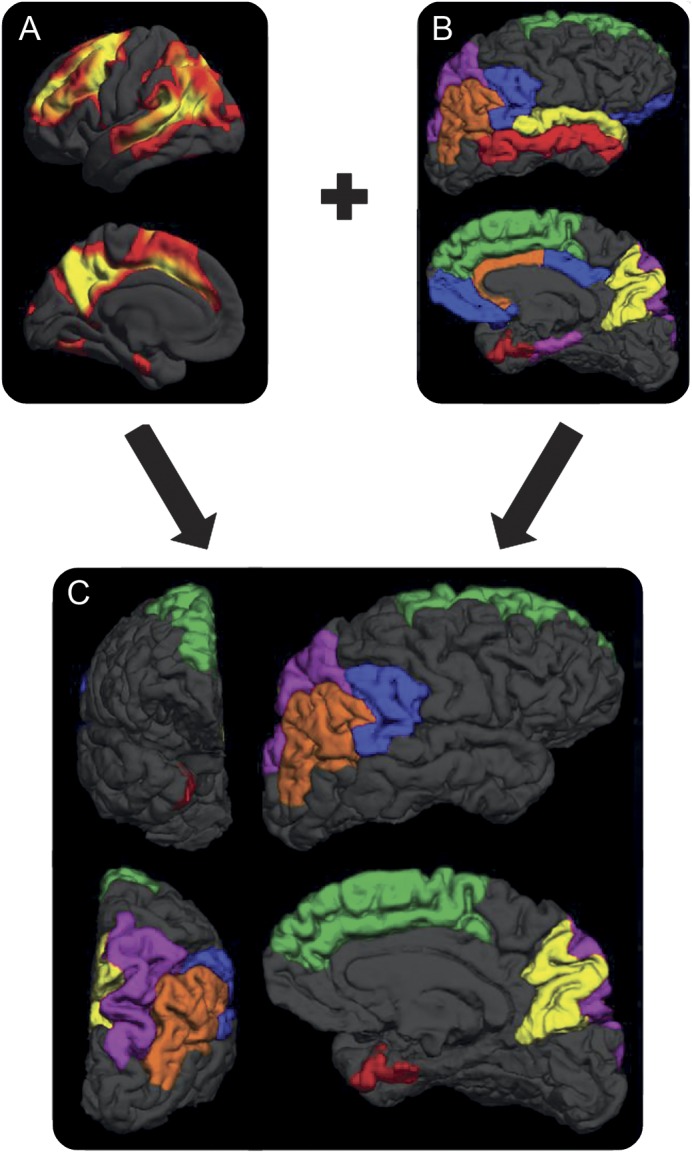
The familial Alzheimer disease (FAD) cortical signature (A) Results of FreeSurfer analysis indicate regions exhibiting the most significant cortical thinning in the mildly symptomatic individuals with FAD from our cohort (*p* < 0.01, family-wise error corrected). (B) The results of a literature search show regions previously highlighted as showing significant atrophy in FAD. We identified regions that were positive in both the literature-driven and data-driven approaches, and included only these regions in our final FAD cortical signature (C). A total of 6 regions were included: entorhinal cortex (red), inferior parietal cortex (orange), precuneus (yellow), superior frontal cortex (green), superior parietal cortex (purple), and supramarginal gyrus (blue).

We first undertook a detailed literature review in PubMed, using the search terms (familial Alzheimer disease) or (autosomal dominant Alzheimer disease) and (cortical thickness) or (cortical volume), to identify the regions previously found to undergo significant cortical atrophy in FAD (appendix e-1, table e-1). In addition, we separately analyzed data from our own cohort, using a conservative statistical threshold (*p* < 0.001, family-wise error corrected), to identify those regions that demonstrated the most significant bilateral thinning in the baseline scans of the 10 mildest (according to MMSE) symptomatic participants (appendix e-1). We then combined the results of these literature-driven and data-driven approaches, and only included regions in the final cortical signature that were identified by both methods (figure 1).

### Longitudinal mixed-effects modeling.

We applied a longitudinal linear mixed effects framework to model cortical thickness in the signature regions using data from all mutation carriers and noncarriers at all available timepoints (appendix e-1). The model included fixed effects for age, sex, mutation carrier status, and polynomial terms for EYO; a family level random effect for intercept; and participant level random effects for intercept, EYO, and mutation carrier status. Of the 85 participants, 55 individuals (33 mutation carriers and 22 controls) had scans at multiple longitudinal timepoints (mean 2.9, maximum 6; mean ± SD interscan interval = 448 ± 172 days). This longitudinal mixed effects approach was used to assess longitudinal change in both absolute cortical thickness and rates of change in cortical thickness.

In addition, we used linear mixed effects models to investigate whether lower baseline thickness was associated with increased rate of subsequent thinning, using data from the 33 mutation carriers who had scans at at least 2 timepoints. In this model, rate of subsequent thinning was estimated by a fixed effect for duration of follow-up. A fixed effect for interaction between baseline cortical thickness and duration of follow-up estimated the strength of association between the observed baseline value and rate of subsequent thinning. The model included a fixed effect for sex, family level random effect for intercept, and participant level random effects for intercept and duration since baseline (appendix e-1).^14^

### Assessing association between cortical signature change and neuropsychological performance.

To assess the clinical implications of thinning within the cortical signature regions, we analyzed associations between cortical thickness and performance on neuropsychological testing in mutation carriers, both before and after symptom onset. Spearman correlation coefficients were used to assess cross-sectional correlation between mean cortical thickness across the 6 signature regions at baseline and score on each focal neuropsychological test. In addition, the following specific correlations were examined, based on hypothesized associations between specific regions and specific focal tests:

1. RMT (words and faces) and entorhinal cortex thickness.
2. Digit span forwards and superior frontal cortex, supramarginal gyrus, and inferior parietal cortex thickness.
3. Digit span backwards and superior frontal cortex thickness.
4. Arithmetic and supramarginal gyrus and inferior parietal cortex thickness.
5. VOSP object decision and supramarginal gyrus, inferior parietal cortex, and superior parietal cortex thickness.

As well as examining the above correlations among all mutation carriers, secondary analyses examined the correlation between cortical thickness and neuropsychological test scores in presymptomatic mutation carriers only. Cluster-adjusted (robust) *p* values were used to account for clustering of individuals within families. All analyses used STATA v13.0 or later.

## RESULTS

### The cortical signature.

Mutation carriers and noncarriers were well-matched for age (43.2 years [SD 9.4] vs 43.9 [9.4]) and sex (15 male/28 female vs 18 male/24 female). The group demographics, with mutation carriers broken down into symptomatic and presymptomatic, are shown in table 1.

**Table 1:**
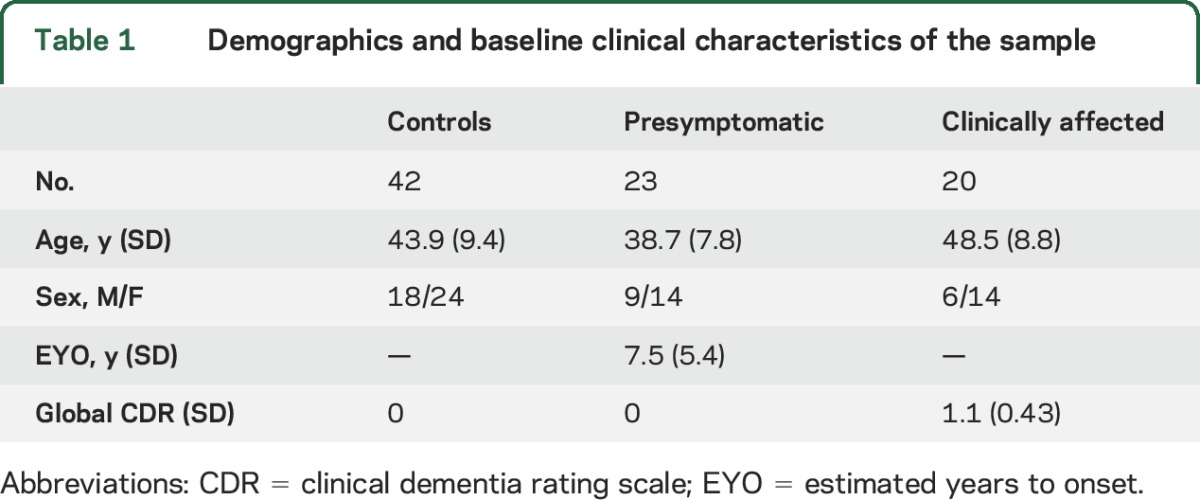
Demographics and baseline clinical characteristics of the sample

Six cortical regions were identified as making up the FAD cortical signature (figure 1): the entorhinal cortex, the inferior parietal cortex, the precuneus, the superior frontal cortex, the superior parietal cortex, and the supramarginal gyrus.

### Presymptomatic cortical thinning detectable across the cortical signature.

**Figure 2:**
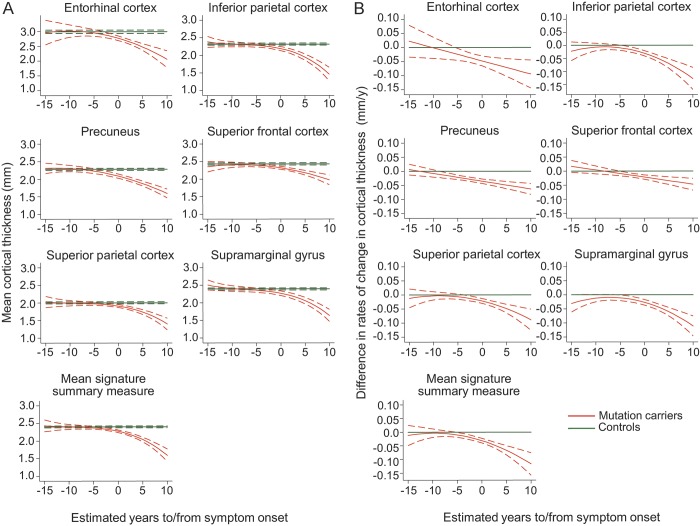
Longitudinal change in cortical thickness in the 6 cortical signature regions Graphs compare mutation carriers and controls for (A) absolute cortical thickness value and (B) difference in rate of change in cortical thickness, with the x-axes representing estimated time to/from onset of progressive cognitive symptoms. Graphs are shown for each cortical signature region as well as the mean signature summary measure. Dotted lines indicate 95% confidence intervals.

Our analysis of longitudinal cortical thickness change found that, for all 6 cortical signature regions, there was strong evidence of a difference in cortical thickness between mutation carriers and controls (*p* < 0.0001 for all regions) and for a difference in rates of change in cortical thickness (*p* < 0.0001 for all regions). Combining all signature regions to give a mean signature measure showed cortical signature thickness to be significantly lower in mutation carriers compared to controls by 3 years before predicted onset (figure 2, table 2). The earliest significant difference in cortical thickness in any individual region was detectable 4 years preonset, in the precuneus.

**Table 2:**
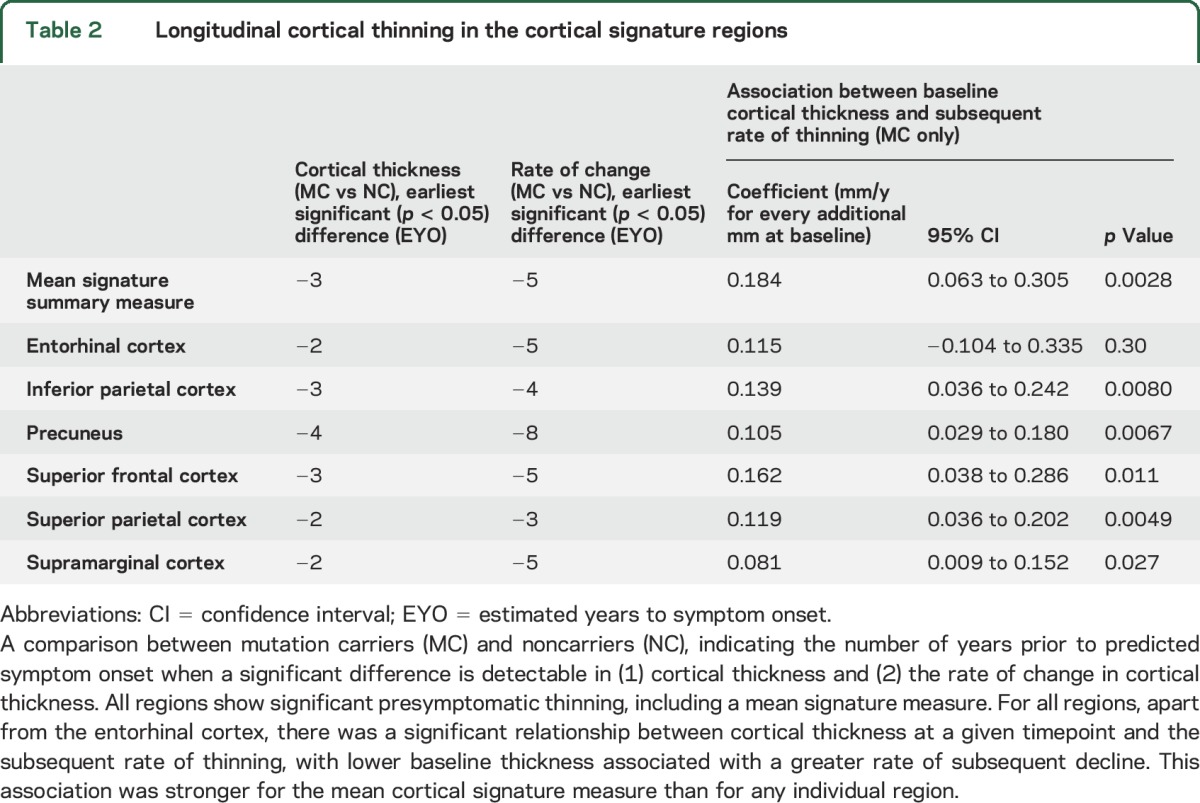
Longitudinal cortical thinning in the cortical signature regions

Earlier differences between mutation carriers and controls were apparent, for all 6 signature regions, when comparing the rate of change in cortical thickness, rather than the absolute cortical thickness value alone (figure 2, table 2). Rates of thinning in the mean signature summary measure become nominally higher in mutation carriers compared with controls at around 9 years before predicted onset, with the difference becoming statistically significant by 5 years before predicted onset. The earliest significant difference in rate of change in an individual region was again observed in the precuneus, with significant differences in rates of thinning seen at 8 years preonset. A paired *t* test, including all 6 signature regions, found the change in rate to be significantly earlier than the change in absolute thickness (*p* = 0.005).

Across all mutation carriers, lower baseline cortical thickness predicted greater thinning in the subsequent follow-up period. This association was stronger for the mean signature summary measure than for any region individually (table 2). A significant association remained between mean signature thickness and future decline even when only presymptomatic carriers were assessed (*p* = 0.022); a similar association was found in this group for the precuneus (*p* = 0.029).

### Correlation between cortical signature thickness and neuropsychological performance.

Across all mutation carriers, there were significant positive correlations between the mean cortical signature summary measure and cognitive performance across tests of a wide range of cognitive modalities (table 3). For each of these tests, there was also significant correlation between test performance and cortical thickness in specific hypothesized individual cortical regions.

**Table 3:**
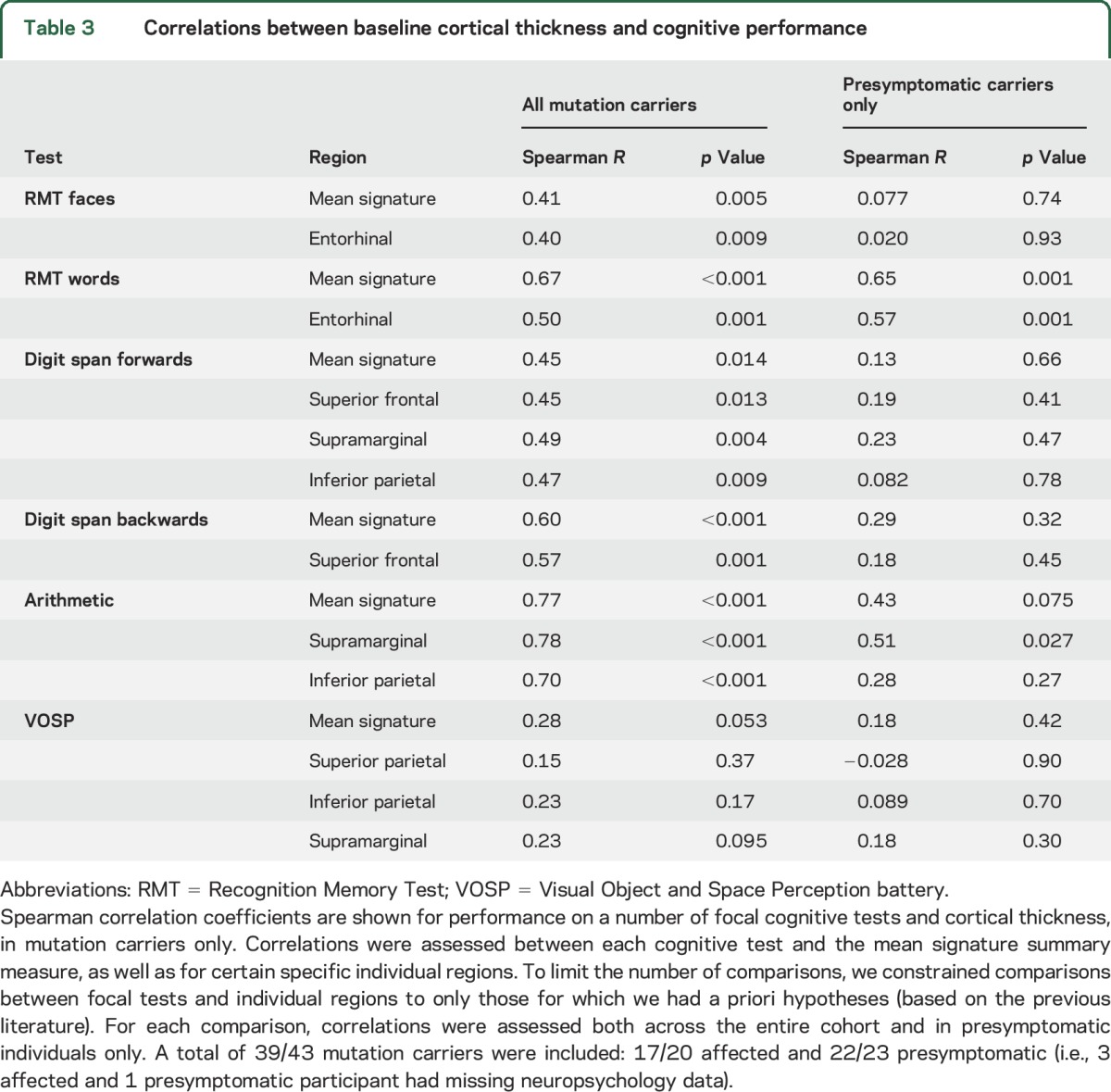
Correlations between baseline cortical thickness and cognitive performance

Looking at the presymptomatic participants only (median time to onset 8 years), there remained a statistically significant correlation between cortical thickness and performance in both verbal recognition memory and arithmetic, but not with other domains (table 3).

Despite the precuneus not being one of our prehypothesized regions for testing associations with cognitive decline, as it had demonstrated the earliest presymptomatic thinning of any individual region, we undertook a post hoc analysis to assess its association with presymptomatic cognitive performance. We found that, in presymptomatic individuals, precuneus thickness correlated with verbal recognition memory only (Spearman *R* = 0.48, *p* = 0.018).

## DISCUSSION

We report results of longitudinal cortical thickness change in presymptomatic FAD within a disease-specific cortical signature. The FAD cortical signature that we identified would appear to closely resemble that previously identified for sporadic AD,^5^ and included medial parietal, lateral parietal, and medial temporal regions. By then applying this cortical signature to an independent group of presymptomatic mutation carriers, we found that all 6 cortical signature regions showed significant thinning a number of years before the onset of cognitive symptoms.

Our findings suggest, consistent with previous studies of FAD mutation carriers, that the precuneus is one of the earliest regions to show significantly reduced cortical thickness, at approximately 4 years preonset.^15,16^ A previous cross-sectional study of FAD, which applied the sporadic AD cortical signature (rather than a FAD-specific one), identified presymptomatic thinning in 3 of the regions identified in our study, including the precuneus, at an average of 6 years prior to onset.^17^ The reason for the slightly earlier precuneus thinning in this study may have been the increased homogeneity of the participants, with all mutation carriers having the same mutation (*PSEN1* E280G).

Two other studies of presymptomatic FAD have reported an initial increase in cortical thickness, prior to later decline.^18,19^ The authors acknowledged that this was unexpected, and speculated that it may indicate a presymptomatic inflammatory process. Here, in a larger sample, we find no evidence to support an early cortical thickness rise. However, the possibility that cortical thickness may increase in the earliest presymptomatic phase—perhaps even earlier than the period our study spanned—before falling as individuals come closer to symptom onset cannot be excluded.

We found, across all 6 signature regions, that the rate of change in cortical thickness allowed significantly earlier detection of neurodegeneration than a cross-sectional measure of thickness at a single timepoint (table 2). Previous imaging studies have similarly suggested that rate of loss rather than absolute volume/thickness may be more sensitive to early pathologic change,^10,11,16,20^ although few FAD studies have measured longitudinal change in cortical thickness specifically. The timing of the increase in rate of decline in cortical thickness from our study was slightly earlier than found for other longitudinal volumetric imaging measures.^11,21^

We found that, in mutation carriers, thickness across the cortical signature regions correlated with cognitive performance (table 3). This was true even prior to the onset of symptomatic decline, in individuals who were on average 8 years from predicted symptom onset. In the presymptomatic mutation carriers, the closest correlation was between cortical thickness and verbal episodic memory. This cognitive domain has previously been identified as being the earliest neuropsychological predictor of later decline.^22–24^ Compared to the precuneus—the cortical region that showed the earliest thinning—the statistical significance of the correlation between mean signature thickness and verbal episodic memory was greater by an order of magnitude.

Our findings also suggest that cortical signature thickness at a given timepoint may have prognostic value, in that it correlates significantly with future rate of decline. When analyzing all mutation carriers, assessing the mean thickness across a composite of preselected cortical regions provided stronger prediction of future decline than any single region alone (table 2), although when looking in presymptomatic individuals only the predictive value of the cortical signature and precuneus were similar. The finding that a multiregion signature provides predictive power and greater correlation with cognitive performance than any region in isolation is consistent with the concept that in AD there is early breakdown of a vulnerable but distributed neural network of different interconnected regions; the variability between individuals in the patterns of loss and sequence of recruitment of different regions with progression means that a composite signature region may be a more robust measure than a single region.^25,26^ A similar cortical signature approach that we use here for FAD has previously shown promise in identifying early symptomatic sporadic AD.^5,6,27^

A composite cortical signature region may be useful in the recruitment to, and monitoring of, presymptomatic trials of disease-modifying therapies.^2,3,7^ The use of a marker of AD neurodegeneration, such as the cortical thickness signature, may allow presymptomatic disease staging by allowing prediction of how close an individual is to symptom onset. A cortical signature could also provide a means of quantifying loss over time, a potentially valuable outcome biomarker.

Compared to previous imaging studies of FAD, our study has a number of novel features that provide important insights into presymptomatic disease. We provide empiric evidence of the value of assessing presymptomatic longitudinal rate of change in cortical thickness, rather than just cross-sectional measurement; of the potential predictive value of using a composite signature region; and that cortical signature thickness is related to cognitive performance even prior to symptom onset.

This study has a number of limitations. In the selection of our cortical signature, we constrained the regions to select from those that had been identified previously in the literature as involved in FAD. While such an approach has the potential to introduce bias, combining a priori information from the literature with an independent dataset can produce more robust results, with greater face validity, when identifying specific features of interest.^28^ In presymptomatic mutation carriers, we used parental age at onset to estimate the age at which an individual will develop cognitive symptoms. While parental age at onset has been shown to correlate closely with actual age at onset and to closely relate to other methods of estimating disease onset (e.g., based on family mean or mutation mean age at onset), this remains a proxy measure,^9^ and it is only with longer term follow-up that the actual age at symptom onset can be confirmed.

A number of different genetic mutations were included in our cohort, including mutations of both the *PSEN1* and *APP* genes. A degree of variability, in terms of both clinical phenotype and radiologic appearance, has been found between the different FAD mutations, both between genes and within the same gene,^29,30^ meaning that our cohort is likely to be heterogeneous. While this diversity means it may be more difficult to identify a common pattern, the inclusion of different mutations and genes means that the composite cortical signature identified is likely to be more robust to interindividual variability. Future validation of the utility of the cortical signature would be valuable, ideally in a large cohort such as the Dominantly Inherited Alzheimer's Network.^31^ Also of interest would be a direct comparison between thinning patterns in familial and sporadic cases.

FAD is characterized by cortical thinning that is detectable presymptomatically. Of the regions studied, the precuneus showed particularly early change. However, a composite signature of multiple vulnerable regions may provide more robust prediction of future decline, and closer correlation with preclinical cognitive change, than measuring a single region alone. Rates of thinning in the cortical signature became significantly abnormal about 2 years earlier than absolute cortical thickness. This imaging measure may have utility in presymptomatic trials.

## Supplementary Material

Data Supplement

## GLOSSARY

AD: Alzheimer disease
CDR: Clinical Dementia Rating
EYO: estimated years to symptom onset
FAD: familial Alzheimer disease
MMSE: Mini-Mental State Examination
RMT: Recognition Memory Test
VOSP: Visual Object and Space Perception battery
