# Supplementary material for: Presymptomatic cortical thinning in familial Alzheimer disease: A longitudinal MRI study
Source: Neurology. 2016 Nov 8;87(19):2050–7. doi: 10.1212/WNL.0000000000003322 (PMC5109950; doi:10.1212/WNL.0000000000003322)
Supplement: Data Supplement [file supp_87_19_2050__index.html]

Presymptomatic cortical thinning in familial Alzheimer disease — Data Supplement 

# Presymptomatic cortical thinning in familial Alzheimer disease

## Data Supplement

**Neurology® data supplements are not copyedited before publication. Published editorials and translations have been copyedited.  
 © 2016 American Academy of Neurology.  
  
 Files in this Data Supplement:**

- Appendix e-1 and Table e-1 - Microsoft Word file
