# Supplementary material for: Presymptomatic cortical thinning in familial Alzheimer disease: A longitudinal MRI study
Source: Neurology. 2016 Nov 8;87(19):2050–7. doi: 10.1212/WNL.0000000000003322 (PMC5109950; doi:10.1212/WNL.0000000000003322)
Supplement: Data Supplement [file supp_WNL.0000000000003322_Appendix_e-1_and_table_e-1.docx]

**Appendix e-1**

**Recruitment of participants**

Individuals were eligible if they had either i) a diagnosis of FAD; ii) a parent affected by FAD; or iii) were a healthy individual with no family history of dementia. With regards to symptomatic FAD participants, efforts were made to recruit controls who were age and gender matched; however in the case of *at-risk* participants from FAD families, for whom we did not know at the point of recruitment if they were mutation positive or negative, recruitment of matched controls was not possible. As FAD is relatively rare, the sample size was primarily limited by the number of mutation carriers that it was possible to recruit within the recruitment period.

**Identifying the FAD cortical signature**

We first undertook a literature review in PubMed, using the search terms [familial Alzheimer’s disease] OR [autosomal dominant Alzheimer’s disease] AND [cortical thickness] OR [cortical volume], to identify articles that had previously examined cortical atrophy in FAD. The title and abstract of all articles identified by the search were assessed for suitability. For all suitable articles (i.e. original studies of FAD with data on cortical volumes/thickness) from the initial search, the titles and abstracts of articles in their reference lists were reviewed, and any additional suitable articles also included. We excluded any articles that included any of the same participants as the current study. A total of seven articles, including a total of 260 FAD mutation carriers, were identified.[^1-7^](#_ENREF_1) Cortical regions identified in these publications as

showing significant differences (p < 0.05) in FAD compared to controls are outlined below in table e-1, as well as in figure 2B of the main manuscript.

| **Study** | **n= (mutation carriers)** | **Cortical regions identified** |
| --- | --- | --- |
| Apostolova et al. | 25 | Superior parietal, inferior parietal, supramarginal, precuneus, posterior cingulate |
| Benzinger et al. | 137 | Superior parietal, inferior parietal, superior temporal, middle temporal, entorhinal, precuneus |
| Fortea et al. | 11 | Banks of superior temporal sulcus, superior parietal, inferior parietal, supramarginal, fusiform |
| Lee et al. | 25 | Superior parietal, superior temporal, middle temporal, superior frontal |
| Quiroz et al. | 18 | Superior parietal, inferior parietal, precuneus |
| Reiman et al. | 20 | Superior parietal, inferior parietal, supremarginal, middle cingulate, superior temporal, parahippocampal, fusiform |
| Sala-Llonch et al. | 24 | Inferior parietal, superior temporal, middle temporal, middle frontal |

**Table e-1 A summary of the cortical regions identified as undergoing significant atrophy in FAD from the 7 studies identified by our literature search**

In addition, we separately analysed data from our own cohort to identify those regions that demonstrated the most significant bilateral thinning in the baseline scans of the 10 mildest (according to MMSE) symptomatic FAD participants (mean age 49.0 (SD 10.8); 7 male/3 female) compared to the 42 controls (43.9 (9.4); 24 male/18 female). The 10 mildly affected participants had a mean CDR of 0.75 (SD 0.26) and MMSE of 24.1 (2.4). Freesurfer was used to measure cortical thickness in these individuals as described in the methods section of the main manuscript. An un-biased whole-brain analysis provided mean cortical thickness for 34 parcellated cortical regions bilaterally. Correcting for age and gender, we used a conservative statistical threshold (p<0.001 FWE corrected) to identify those cortical regions with evidence for significant thinning compared to controls (figure 2A).

We purposely chose a relatively liberal threshold for the initial literature search, to identify all regions that have previously been implicated as undergoing significant atrophy in FAD, prior to then applying much more stringent statistical criteria to the data-driven analysis that followed.

Comparing the results of the literature-driven and data-driven approaches, and including only regions identified by both approaches, we identified consistent cortical regions showing thinning in FAD (figure 1C), which we used to determine the final FAD cortical signature.

**Longitudinal mixed effects modelling**

We applied a longitudinal linear mixed effects framework to assess longitudinal change within the cortical signature regions, in terms of both absolute cortical thickness and rates of change in cortical thickness, using data from all mutation carriers (symptomatic and presymptomatic) and non-carriers at all available time-points.

The outcome (dependent variable) was cortical thickness in the region of interest in mm. The fixed effects predictor variables were age in years, gender, mutation carrier status, and polynomial terms for years from symptom onset (EYO) in mutation carriers only. All models included a linear and quadratic term for EYO. A cubic term for EYO was also included if there was evidence that this provided a better model fit (p<0.05 Wald test), which was the case for the mean signature summary measure, inferior parietal cortex, superior parietal cortex and supramarginal gyrus.

Random effects included a family level random effect for intercept; and participant level random effects for intercept and EYO in all models with independent variance-covariance structure. A random effect for mutation carrier status was included if there was evidence that this provided a significantly better fit (p<0.05 likelihood ratio test), which was the case for inferior parietal cortex; precuneus and supramarginal gyrus. The random effects for years to onset and mutation carrier status had unstructured variance-covariance, to allow for a correlation between these two random effects.

We applied longitudinal mixed models to investigate the association between the observed baseline thickness and subsequent post-baseline rate of thinning, based on methods outlined by Byth and Cox.[^8^](#_ENREF_8) These models were restricted to the data from the 33 mutation carriers for with scans on at least 2 time-points. The outcome (dependent variable) was cortical thickness in the region of interest in mm. The fixed effects predictor variables were duration of follow-up, gender, interaction between duration and gender, and interaction between baseline cortical thickness (in mm) and duration. Random effects included a family level random effect for the intercept, and participant level random effects for intercept and duration with unstructured variance-covariance.

**References**

1. Apostolova LG, Hwang KS, Medina LD, et al. Cortical and hippocampal atrophy in patients with autosomal dominant familial Alzheimer's disease. Dement Geriatr Cogn Disord 2011;32:118-125.

2. Benzinger TL, Blazey T, Jack CR, Jr., et al. Regional variability of imaging biomarkers in autosomal dominant Alzheimer's disease. Proc Natl Acad Sci U S A 2013;110:E4502-4509.

3. Fortea J, Sala-Llonch R, Bartres-Faz D, et al. Increased cortical thickness and caudate volume precede atrophy in PSEN1 mutation **.**carriers. J Alzheimers Dis 2010;22:909-922.

4. Lee GJ, Lu PH, Medina LD, et al. Regional brain volume differences in symptomatic and presymptomatic carriers of familial Alzheimer's disease mutations. J Neurol Neurosurg Psychiatry 2013;84:154-162.

5. Quiroz YT, Stern CE, Reiman EM, et al. Cortical atrophy in presymptomatic Alzheimer's disease presenilin 1 mutation carriers. J Neurol Neurosurg Psychiatry 2013;84:556-561.

6. Reiman EM, Quiroz YT, Fleisher AS, et al. Brain imaging and fluid biomarker analysis in young adults at genetic risk for autosomal dominant Alzheimer's disease in the presenilin 1 E280A kindred: a case-control study. The Lancet Neurology 2012;11:1048-1056.

7. Sala-Llonch R, Llado A, Fortea J, et al. Evolving brain structural changes in PSEN1 mutation carriers. Neurobiol Aging 2015;36:1261-1270.

8. Byth K, Cox DR. On the relation between initial value and slope. Biostatistics 2005;6:395-403.
